# The Application of Three-Dimensional Technologies in the Improvement of Orthopedic Surgery Training and Medical Education Quality: A Comparative Bibliometrics Analysis

**DOI:** 10.3389/fbioe.2022.852608

**Published:** 2022-03-22

**Authors:** Jian Shi, María José Cavagnaro, Shaokang Xu, Mingyi Zhao

**Affiliations:** ^1^ Department of Spine Surgery, The Third Xiangya Hospital, Central South University, Changsha, China; ^2^ College of Medicine-Phoenix, The University of Arizona, Phoenix, AZ, United States; ^3^ Xiangya School of Medicine, Central South University, Changsha, China; ^4^ Department of Pediatric, The Third Xiangya Hospital, Central South University, Changsha, China

**Keywords:** three-dimensional technologies, surgical simulation and training, orthopedics, bibliometrics analysis, 3D printing, methodology, education and patients counseling

## Abstract

Orthopedics is a medical specialty that focuses on the clinical treatment and care of the musculoskeletal system. Orthopedics is a medical specialty which specializing in the clinical treatment and nursing of musculoskeletal system. The education of orthopedics is often serious and difficult because of the high technical requirements, complicated anatomical knowledge and long study process. However, medical students or junior residents rarely have the opportunity to see such orthopedic surgery or attend preclinical practice, which limits the opportunities for training clinicians. Hopefully, with the increasing use of three-dimensional (3D) technologies in medical teaching, this situation can be alleviated. In this study, we demonstrate that different 3D technologies can effectively simulate orthopedic surgery with very high accuracy. We carefully evaluated the use of 3D technologies in primary medical teaching and proposed a vision for the future. We searched and screened 3,997 publications from the Web of Science Core Collection (WoSCC) on 22 Oct 2021 with (trauma) AND ((education) OR (training) OR (teaching) OR (learning)) AND ((3D) OR (Three Dimensional)), (Joint) AND ((education) OR (training) OR (teaching) OR (learning)) AND ((3D) OR (Three Dimensional)), (spine) AND ((education) OR (training) OR (teaching) OR (learning)) AND ((3D) OR (Three Dimensional)) as the search strategy. Then, we eliminated the publications irrelevant to “orthopedics” AND/OR “orthopaedic” (in United Kingdom English), the final number of publications are 440 for trauma surgery, 716 for joint surgery and 363 for spine surgery, a visual display of comprehensive information analysis was made by VOSviewer. Next, we read and analyzed retrieved articles extensively according to the selection criteria, 11 highly cited publications on three major branches of orthopedics were chosen. The extracted data included the authors, purpose, methods, results and benefits/limitations. The evaluation of these studies directly and objectively proved the superiority of 3D technologies in orthopedics. Furthermore, the material usage and strength of 3D technologies can be closer to the real situation, which will help improve their effectiveness in teaching. We hope that more relevant studies will be conducted to continue examining the effects of 3D technologies on orthopedic medical education as well as orthopedic surgery training, and we hope that this technique can be more widely used in the clinical teaching of orthopedics to train clinicians on learning medical theory and surgical technology quickly and efficiently.

## Background

Three-dimensional (3D) technologies, in both the digital and physical environments, are playing a more significant role in society than ever before (Treleaven and Wells, 2007; Whitaker, 2014; Awad et al., 2018). With the rapid development of 3D technologies, different forms are also increasing, such as 3D printing models, 3D virtual software, 3D navigation systems and so on. These new 3D technologies are giving an impetus to the change of medical education pattern, especially for orthopedic medical education (Cartellieri et al., 2001; Sharkawi et al., 2008; Oshiro and Ohkohchi, 2017). More and more teaching hospitals and medical colleges are fitted out with the equipment with the latest 3D technologies. Whether it is virtual software or printed models, the technologies have been increasingly used in orthopedic medical education as well as clinical teaching practice. In the field of trauma surgery, joint surgery and spine surgery which are the three most important branches of orthopedics, 3D technologies were also widely and extensively used (Papagelopoulos et al., 2018). 3D technologies may have incomparable advantages compared with traditional 2D-based medical teaching, it could not only better realize the transmission of spatial conformation but also allow students to learn in an interactive environment which would be likely to improve the learning performance and personal satisfaction.

Bibliometrics is a novel discipline that uses a variety of statistical methods to analyze publications, books, documents and articles (Broadus, 1987). It can qualitatively and quantitatively analyze the literature from multiple dimensions, such as co-citation analysis, co-author analysis, bibliographic coupling and co-occurrence analysis, and usually involve deep cluster analysis. The most basic foundation of bibliometrics is to construct the citation graph which is a detailed network or diagram representation of the citations between different publications (Klein and Bloom, 2005; Kokol et al., 2021). In addition, visualization is also an important attribute of bibliometrics. By integrating complex and incomprehensible data and presenting them in the form of images, researchers can quickly and clearly obtain the latest research hotspots and directions, so as to provide a theoretical basis for further research. Bibliometrics is based on large-scale literature databases for analysis, and has become a hot research method in many fields. In this study, we use VOSviewer, an important bibliometric tool developed by Nees Jan van Eck and Ludo Waltman at Leiden University’s Centre for Science and Technology Studies (CWTS), to perform the above bibliometric tasks to complete the above work of literature analysis (Van Eck and Waltman, 2013).

To the best of our knowledge, there is no systematical and specialized research on the application of 3D technologies in the field of orthopedic medical education by using bibliometric analysis methods. However, a growing number of related research in this field, especially in 3D printing models and 3D virtual software have appeared in recent years. Therefore, the purpose of this study is to make real-time analytics of the existing relevant research and present contemporary trends of the connections and relationships between 3D technologies and orthopedic medical education, so as to find the current research hotspots and lay a theoretical foundation for the following educational research. We also seek to clarify the efficiency of the use of 3D technologies in medical education. We hope that this review clarifies the future development direction of the 3D technologies and orthopedic medical education-related research.

## Materials and Methods

### Search Strategies

Data were downloaded from the Science Citation Index-Expanded database of the Web of Science Core Collection (WoSCC) on 22 Oct 2021. We searched from three different aspects which are trauma, joint and spine respectively with the following search strategy: (trauma) AND ((education) OR (training) OR (teaching) OR (learning)) AND ((3D) OR (Three Dimensional)), (Joint) AND ((education) OR (training) OR (teaching) OR (learning)) AND ((3D) OR (Three Dimensional)), (spine) AND ((education) OR (training) OR (teaching) OR (learning)) AND ((3D) OR (Three Dimensional)). The number of publications retrieved was 688(trauma), 2,797(joint) and 512 (spine). Next, we eliminated the publications irrelevant to “orthopedics” AND/OR “orthopaedic” (in United Kingdom English), the final number of publications is 440, 716, and 363. Then we exported all the original data, including year of publication, language, journal, title, author, affiliation, keywords, document type, abstract and counts of citation which were exported into CSV (comma-separated values) format. Next, we read and analyzed retrieved articles extensively according to the selection criteria, 11 highly cited publications with greater academic influence on three major branches of orthopedics were chosen. The extracted data included the authors, purpose, methods, results, and benefits/limitations.

### Data Processing

In the process of data processing, VOSviewer (version 1.6.17) was used to analyze all keywords, countries, years of publication, organizations, and journals. All the results are presented visually. In addition, through extensive reading of all the publications, we chose a few of the most important and relevant publications in each category for detailed analysis and discussion.

## Results

**FIGURE 1:**
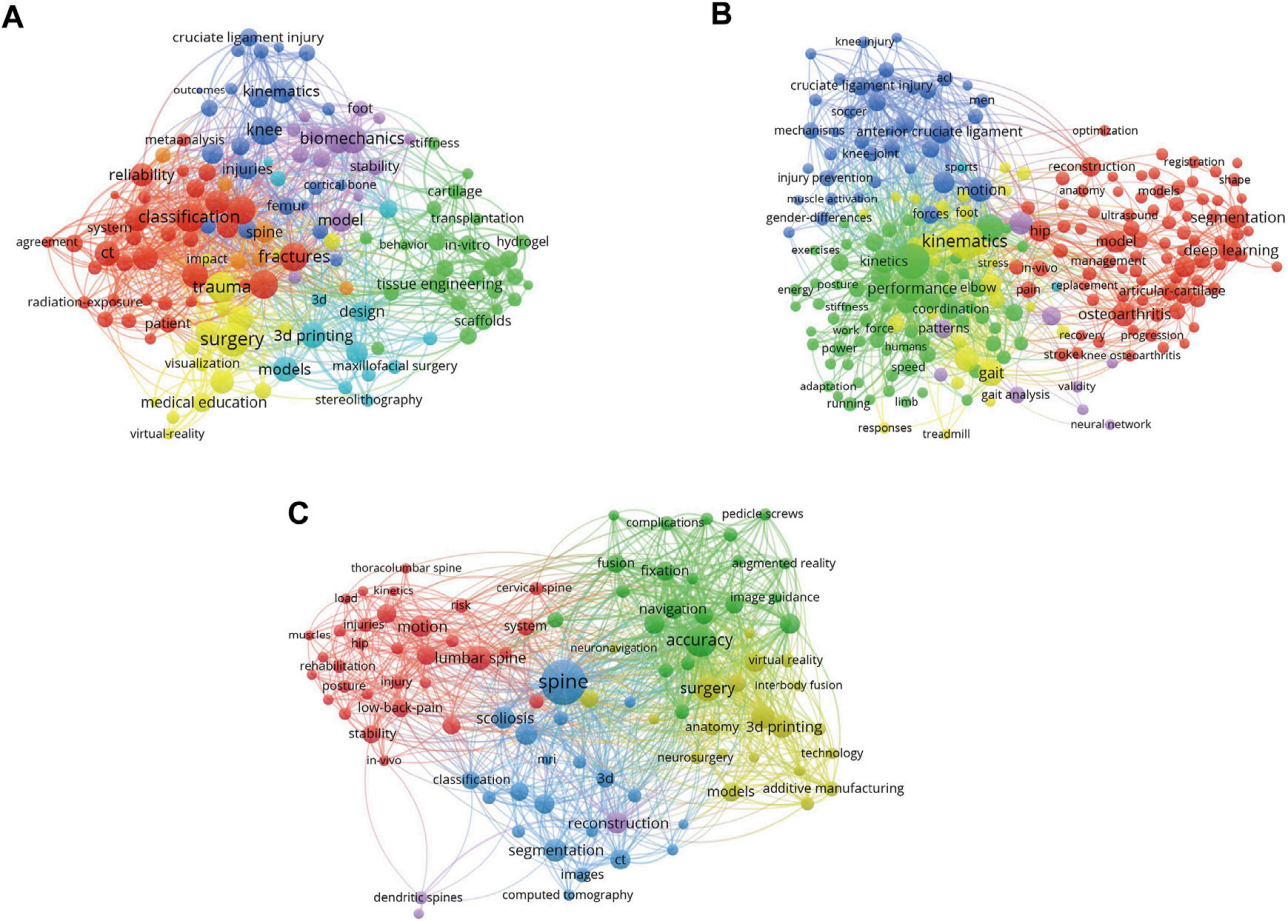
Visualized images of keywords clustering from different sections. **(A)** The all keywords of trauma surgery related publications. **(B)** The all keywords of joint surgery related publications. **(C)** The all keywords of spine surgery related publications. (The correlation between the two keywords refers to how many publications the two keywords have appeared together. Here, the more links between the two bubbles, the more publications they appear together, and the stronger the correlation. In addition, we conduct cluster analysis according to the correlation of keywords, and divide all keywords into multiple clusters. Bubbles in the same cluster have the same color, that is, they have stronger correlation.)

The data was assessed and synthesized according to predetermined and explicit search strategies. According to the common and shared keywords, cluster analysis is carried out and divided into several clusters. In each figure, different colors represent different clusters. Therefore, bubbles of the same color represent a stronger correlation. Figure 1 demonstrates the features of applications in medical teaching from three major disciplines of orthopedics (trauma, joint, and spine surgery). Links between circles represent the frequency of their common occurrences, and the frequency of common occurrence between keywords of the same color is relatively higher. In the trauma section (Figure 1A), multiple essential keywords are shown, which suggest that maxillofacial surgery, cortical bone and femur are recently the research hotspot. And in the joint section (Figure 1B), osteoarthritis, anterior cruciate ligament shows the important position while in the spine section (Figure 1C), pedicle screws, lumbar spine, low-back-pain and scoliosis have become the new trend and show increasingly great importance in the field of 3D technologies application in orthopedic medical education.

**FIGURE 2:**
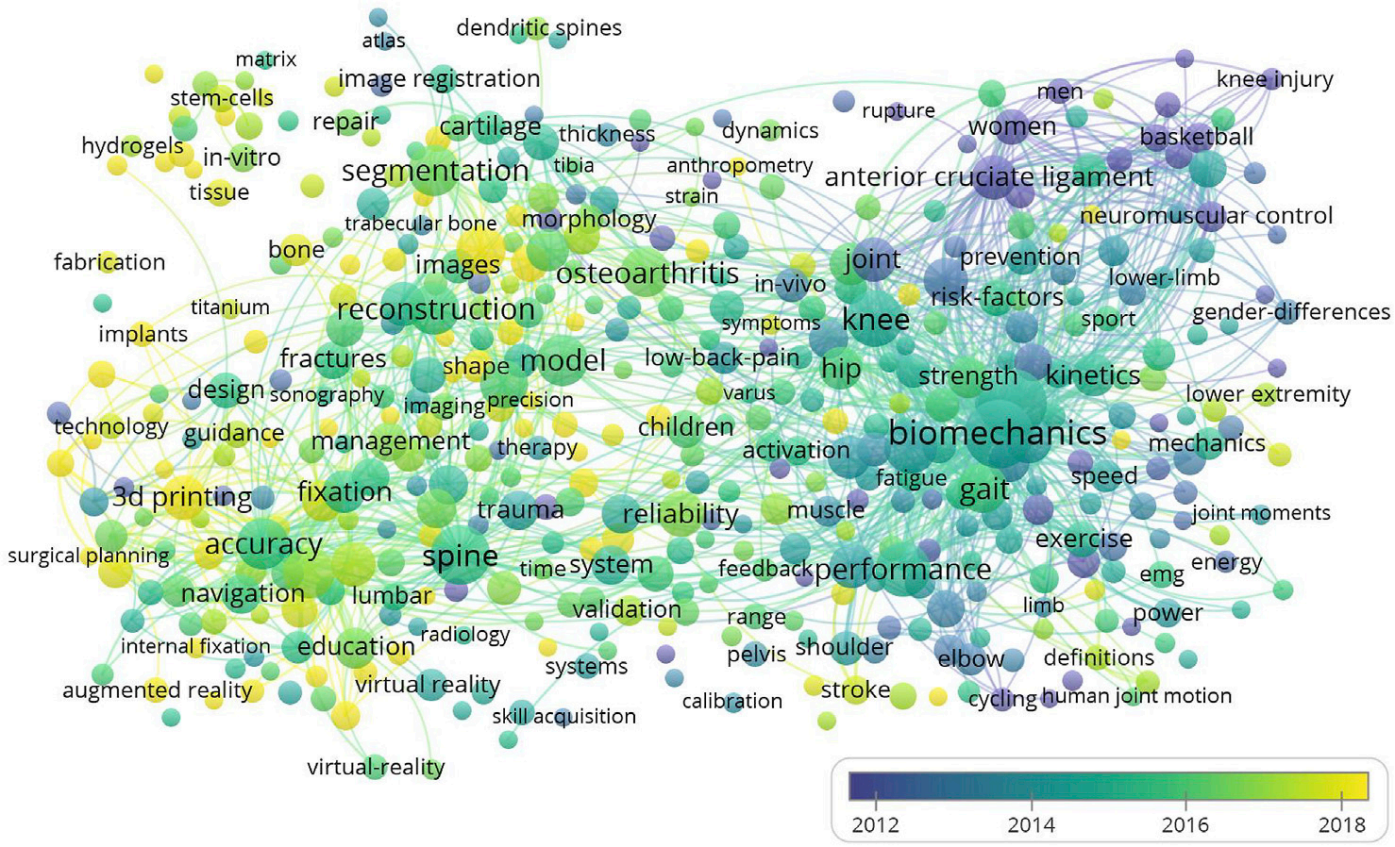
Visualized images of all keywords clustering. (Larger bubbles represent a higher number of occurrences of the keyword in these publications. There are more connections between bubbles closer in space, which means they have a higher chance of appearing together in a publication. In addition, bubbles with brighter colors represent a higher occurrence rate in recent publications, while dark bubbles tend to appear in past publications).

Then a comprehensive analysis combined with major branches of orthopedics was performed. Figure 2 combines all the important keywords in the publications that related to the application of 3D technology in orthopedic medical education. Different colors show different time periods, and blue bubbles represent the research hotspots in the past, and yellow bubbles represent the hotspots in recent years. There are much more publications in research fields such as biomechanics, gait, cruciate ligament and joint coordinate system. Although there are few types of research on implants, stem cell, spine surgery, they belong to a relatively new period, which could be very potential educational research as well as orthopedic surgery training directions in the future.

**FIGURE 3:**
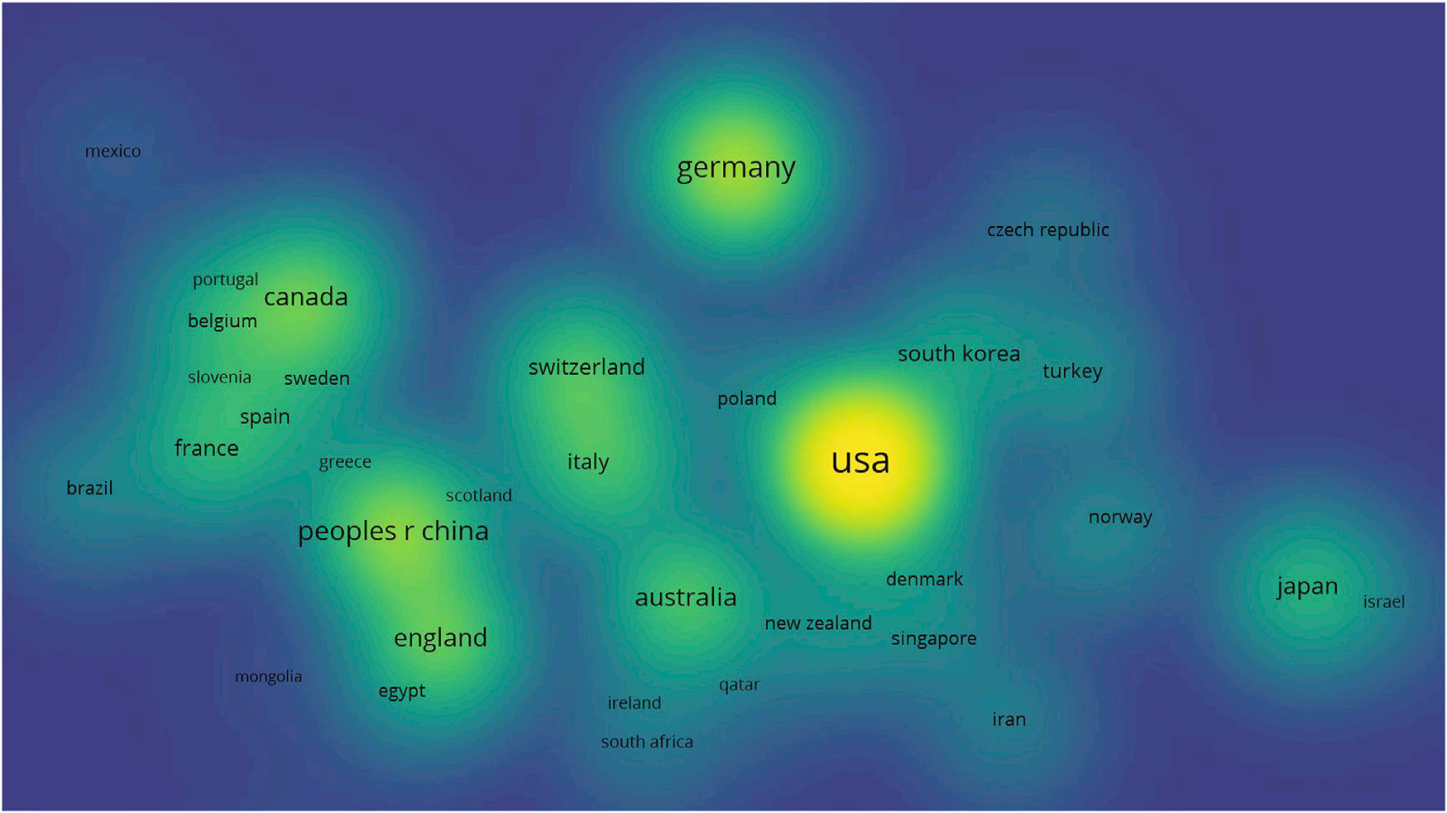
Density visualization of the countries’ distribution of all the publications. (Analyzing is based on the co-authorship of the countries, spatially closer bubbles show that they are close to the same cluster during clustering, which proves that their cooperation relationship is closer. In addition, the brighter the bubbles represent a higher number of publications of the country in the field of 3D technology in orthopedics education).

**FIGURE 4:**
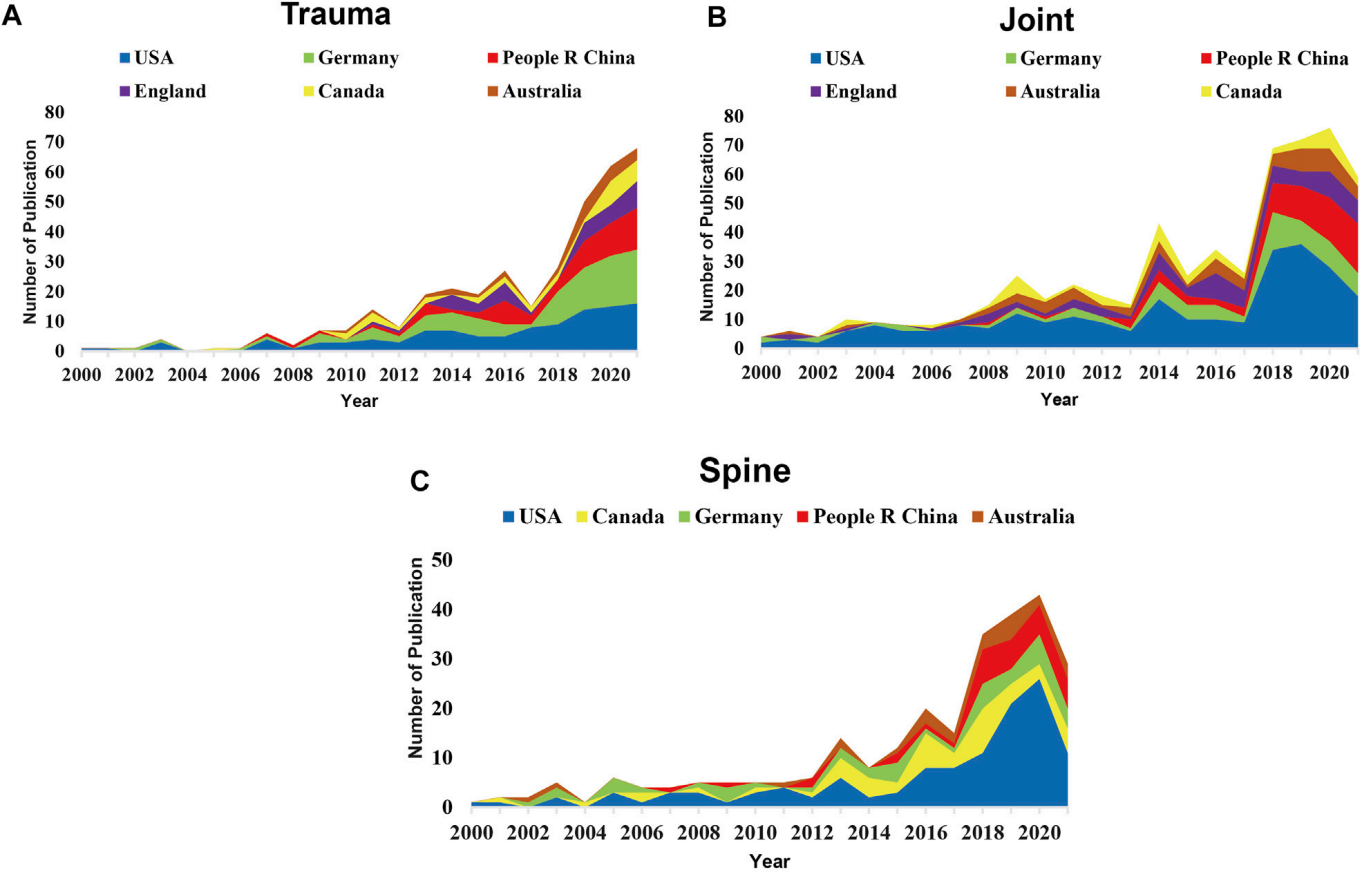
Visualization of number of publications for the top ranked countries. **(A)** The annual publication data of the top countries in terms of application of 3D technology in trauma surgery education and training. **(B)** The annual publication data of the top countries in terms of application of 3D technology in joint surgery education and training. **(C)** The annual publication data of the top countries in terms of application of 3D technology in spine surgery education and training. (Cumulative figure of the number of published articles in the top countries in the three fields of trauma, joint and spine from 2000 to 2021, which reflects the in-depth research of each country in the three fields).

Next, the detailed information of countries of the publications is analyzed. We found that most of the publications were from the United States, Germany, China, England, and Canada (Figure 3). Brighter and larger patterns indicate more publications from a certain country. The number of publications in other developing countries was relatively small. The most productive country, the United States has the largest number of participants from other countries, that is, it has more cooperation with other countries. The number of articles published by developing Asian countries like Turkey and Iran is also relatively small. Figure 4 shows the annual publication data of the top countries in terms of literature publication, from where we could find that the overall trend of publications on orthopedic medical education is increasing comparatively fast in all sections. In addition, countries have made slightly different progress in these three orthopedic branches. In fact, when we conduct in-depth research in different fields, we can choose the paper of the corresponding country for in-depth reading.

**FIGURE 5:**
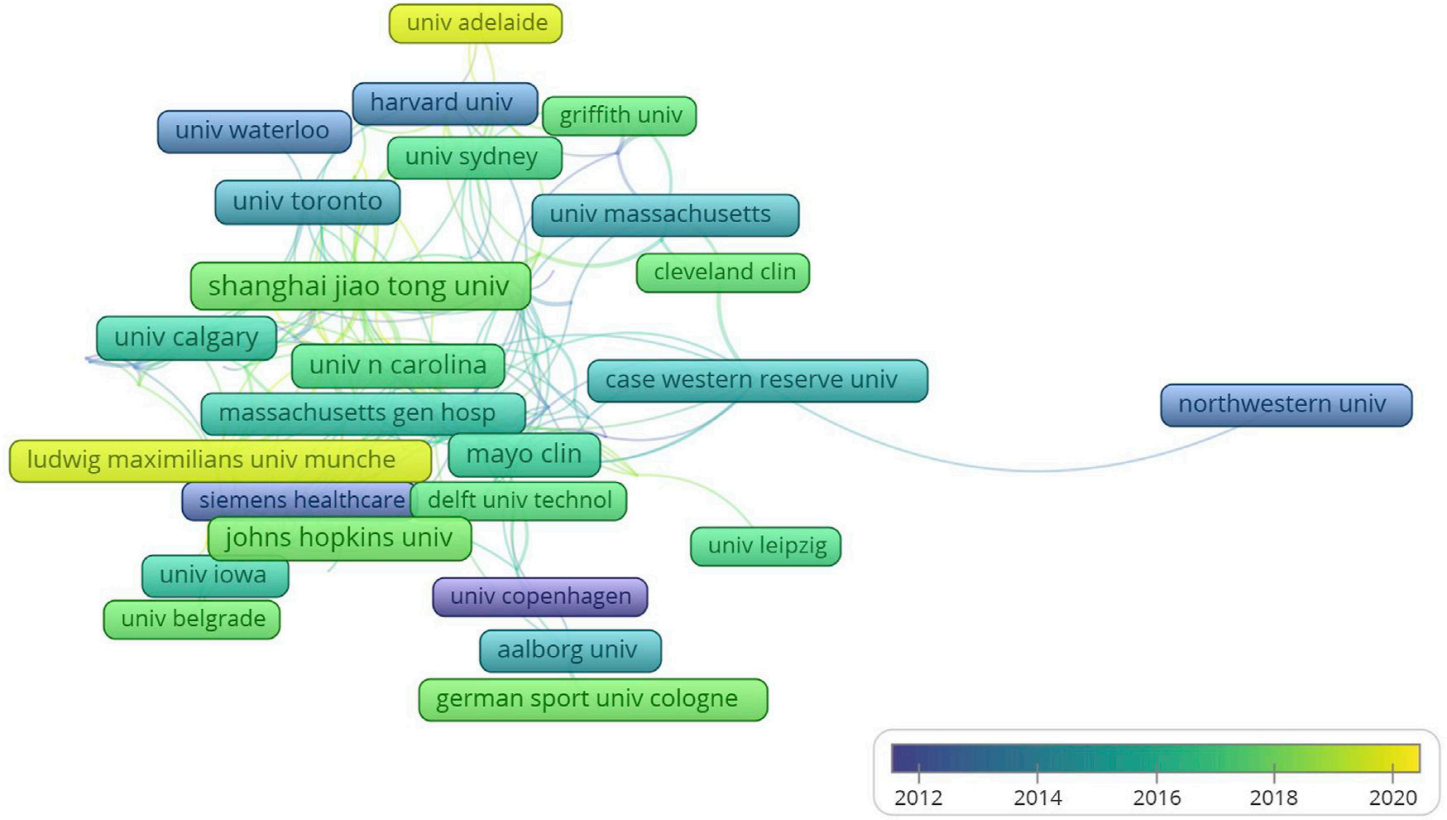
Visualization of institutional cooperation and publication period. (The links between the boxes reflects the number of cooperation, and the closer it is arranged in space, the deeper its cooperation relationship is. In addition, in terms of time, the brighter the color, the more research results the institution has made in recent years, and the darker the color, the more contribution it may have made in the past).

**FIGURE 6:**
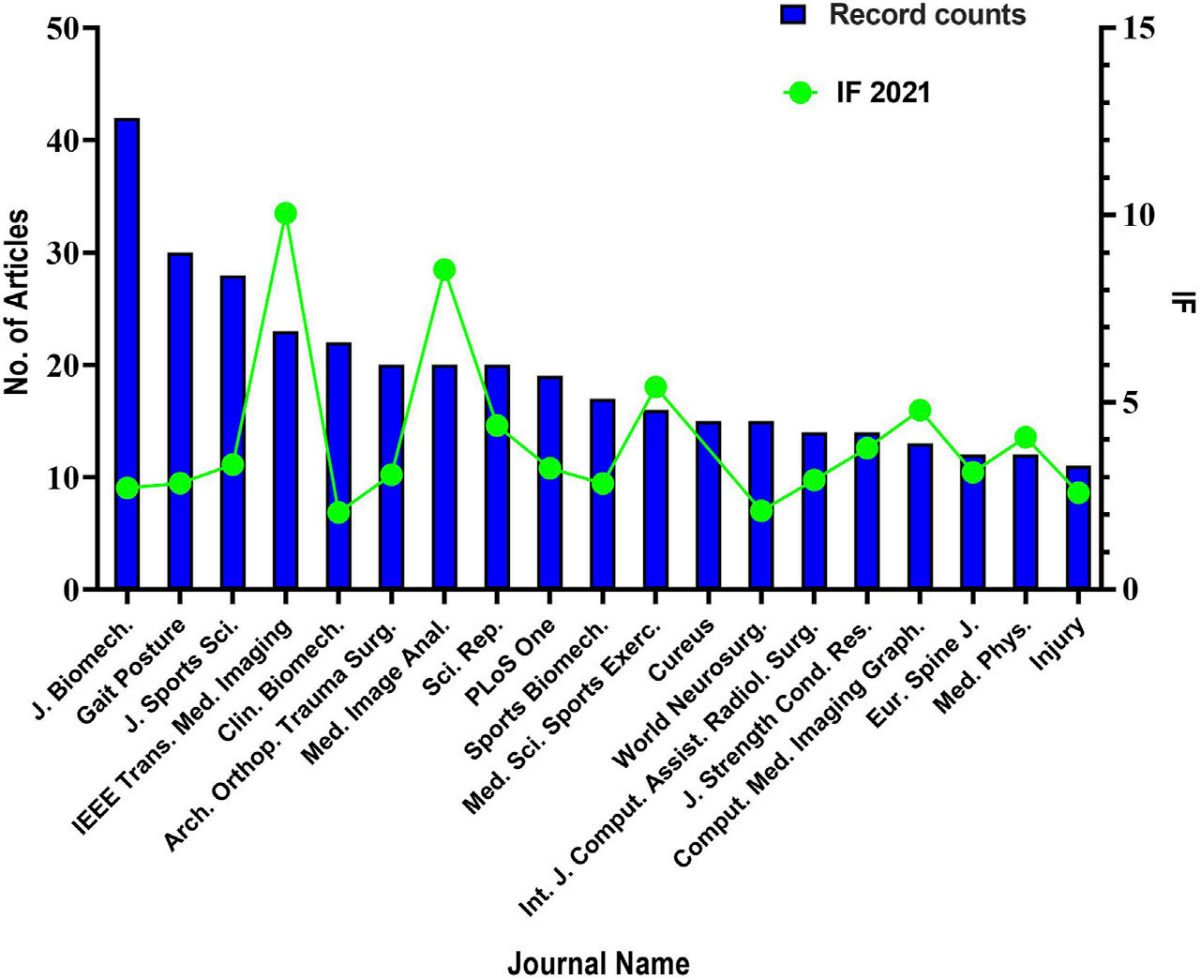
Combinated visualization of number of publications and impact factor for the top-cited journals. (The bar chart reflects the number of publications, and the line chart reflects the impact factors. According to statistics, among all publications sources, these journals have a number of publications published which is no less than 11).

The institutions with the largest number of publications were led by Johns Hopkins University, Shanghai Jiao Tong University, Harvard University, University of North Carolina System, and so on (Figure 5). The link line in the figure refers to the cooperation between different institutions, brighter colors indicate more recent publications while darker colors indicate less recent publications. More connecting lines signified a tighter cooperation relationship between different institutions. In a word, when reading articles, you can focus on selecting institutions that have made outstanding contributions in the near future, such as Ludwig Maximilian Muenchen Unitversitaet. Figure 6 enlist the journals of the top-cited publications in descending order by the impact factor and the average number of citations per paper (2021/last 5 years). From where we could find that Journal of Biomechanics, Gait and Posture and Journal of Sports Sciences were the top three. And a large proportion of the journals focus on the basis of the combination of medical science with engineering technology. These imply that the importance of interdisciplinary studies in 3D technologies-based orthopedic medical education research. By presenting the number of publications and the if value of journals, readers can select journals close to the subject and of good quality, and focus on reading, which may be helpful to their cognition in this field.

## Discussion

For many years, the teaching method for medical students has remained at the level of flat images and two-dimensional level, cadaver specimens or three-dimensional models often require high costs (Erolin, 2019), there are also advanced courses for prospective specialists, some of which are offered by companies or by professional societies such as the AO and it is similarly difficult for ordinary medical students or teaching institutions to afford. However, only two-dimensional elements are not enough for most people to understand and master the complex spatial structure. Especially for the teaching of orthopedics, which has complex spatial structure and requires a lot of spatial conception ability, traditional teaching methods have many limitations (Moro et al., 2017). Therefore, a qualified orthopedic doctor often needs long-term training and a large number of educational resources. With the development of 3D technology, the trend of its application in the field of teaching is also further expanding. Promoting its development in orthopedic education will save a lot of teaching resources and release more human and material resources to invest in unknown scientific fields.

As one of the most rapidly developing technologies, 3D technologies have played an increasingly important role in the development of medical science like 3D printing or 3D navigation. Through the digital synthesis of professional 3D software, two-dimensional elements or flat images can be transformed into three-dimensional conformation, and then presented in the form of interactive software or printed model, so that people can understand their spatial structure without real objects (Nam et al., 2012). Current mainstream 3D technologies include 3D printing models, 3D virtual software, 3D navigation systems and so on (Treleaven and Wells, 2007; Whitaker, 2014; Awad et al., 2018). With the renewal of materials and the development of computer technology, 3D models are much closer to real objects and have a high degree of reduction. Compared with human specimens, both 3D virtual images and 3D printed models are less vulnerable to damage and have lower transmission costs, which makes them available to some remote areas or primary medical institutions. In addition, holographic projection technology is also maturing. We’ve already seen a number of tremendous changes and improvements in orthopedic medical education brought about by different 3D technologies. Instead of displaying the different visual bibliometrics results from multiple perspectives which have already been shown in the result section, we also read and evaluate all the retrieved articles in detail extensively, 11 representative publications from three major branches of orthopedics were chosen (Van Sint Jan et al., 2003; Podolsky et al., 2010; Gottschalk et al., 2015; Park et al., 2018; Mishra et al., 2019; Wang et al., 2019; Montgomery et al., 2020; Clifton et al., 2021; Foo et al., 2021; Kim et al., 2021; Papavasiliou et al., 2021), comprehensive evaluative analysis was made from basic information, purpose, assessments, results, benefits and limitations (Table 1, trauma surgery *n* = 4; Table 2, joint surgery *n* = 3; Table 3, spine surgery *n* = 4).

**TABLE 1:** Trauma.

Paper authors	Purpose	Assessments	Results	Benefits	Limitations	Year
Huixiang Wang et al. Wang et al. (2019)	Compare the efficacy of 3D interactive software with traditional 2D in learning acetabular fracture classification	30 junior doctors were randomly but equally allocated to two groups: experiment and control, the experimental group was required to operate the 3D software to observe, while the control group learned through the traditional 2D atlas. Both groups were then tasked to classify ten acetabular fractures and complete a five-point Likert scale on their satisfaction of each learning modality	The experimental group significantly outperformed the control group (t (28) = 2.526, P = 0.017) with identifying correct acetabular fracture classification	The 3D software has improved classification accuracy and higher Likert scale score	There is confusion in the presentation of 3D images	2019
Spencer Jason Montgomery et al. Montgomery et al. (2020)	To determine if 3D printed calcaneal fracture models can improve orthopedic trainee education	16 resident trainees and 5 attending surgeons alternated between 2 computer stations to complete a total of 10 stations (5 with CT and 3Dp model vs. 5 with CT and no 3Dp model). They rated their level of confidence in their understanding of the injury and rated their perceived accuracy of the 3Dp model or 3D volume rendering using standardized visual analogue scales	Perceived accuracy of cases with 3Dp models was significantly higher than cases without 3Dp models (7.0 vs. 5.5 *p* < 0.001)	3Dp models increase the perceived accuracy of fracture assessment	No statistically significant improvement in diagnostic accuracy was observed	2020
Abhishek Mishra et al. Mishra et al. (2019)	Assess the worth of 3D printing in virtual preoperative planning (VPP) and designing various models	Create virtual 3D models and 3D printed models for some of the 91 cases before surgery and investigate the assessement of the surgeons. Then the surgeons were asked to finish a questionnaire	The surgeons were satisfied with the outcome. Surgical time was reduced, with a better outcome. It’s helpful in understanding the anatomy and sketching out the plans for optimum reduction andfixation	3DP is useful in complex trauma management by accurate reduction and placement of implants, reduction of surgical time and with a better outcome	There is an initial learning curve to understand and execute the 3D printing	2019
Theodora Papavasiliou et al. Papavasiliou et al. (2021)	Explore the impact of 3D printed *ex vivo* hand models on trainees’ skills	There were 20 surgery residents become the trainees. Evaluate the trainees’ pre and post skills by using a score system (global rating scale) in the beginning and at the end of the module	The overall average scores of the cohort before and after assessment were 23.75 and 34.7, respectively. Significant (*p* < 0.01) difference of improvement of skills was noted on all trainees	Helped trainees improve their skills with regard to K-wire fixation techniques, including improvement of their understanding of the 3D bone topography	No published	2021

**TABLE 2:** Joint.

Paper authors	Purpose	Methods	Results	Benefits	Limitations	Year
Chung Yoh Kim et al. Kim et al. (2021)	Presented movable surface models to help medical students understand the multiaxial movements of the hip joint	The muscle and bone surface models were moved over six movements of the hip joint (flexion, extension, abduction, adduction, lateral rotation, and medial rotation)	It enabled users to see the stereoscopic shapes of the bones and muscles of the hip joint and to scrutinize the six movements on the X, Y, and *Z* axes of the joint	It will be helpful for medical students to learn the multiaxial movements of the hip joint	No published	2021
Q. C. Foo et al. Foo et al. (2021)	A 3d temporomandibular joint (tmj) prototype was developed to the training of arthrocentesis and arthroscopy	Use professional materials and 3d printing technology to produce mandible and skin tissue	A comparison of various teaching techniques including cadaver and synthetic joint models concluded that the cadaver-based teaching method was superior	The cost of printing the model is very low	Structural weakness. In addition, not reproduce the tensile strength and elasticity of the human tmj capsule. Forward sliding of the condyle and anterior movement of the articular disc could not be replicated	2021
Serge Van Sint Jan et al. Van Sint Jan et al. (2003)	A method aiming to the creation of a fully interactive 3d environment allowing joint simulation was reported in this paper	The joint physiological parameters were measured and collected, and the 3d conformation was obtained by ct scan. Then the 3d reconstruction was carried out to make the virtual software	A 3d environment which can maximize joint simulation	It is based on fully experimental data, to ensure that the results are physiologically correct. The joint simulations obtained can be integrated into anatomy lectures	No published	2003

**TABLE 3:** Spine.

Paper authors	Purpose	Assessments	Results	Benefits	Limitations	Year
Hyun Jin Park et al. Park et al. (2018)	Evaluate the educational effect of using a real-size 3D-printed spine model for training beginners of the free-hand pedicle screw instrumentation technique	2 novice surgeons who had no experience of free-hand pedicle screw instrumentation technique were asked to insert 10 pedicle screws for each lumbar spine model. The accuracy and duration of the operation will be recorded. The results of the latter 10 spine models were compared with those of the former 10 models to evaluate learning effect	The latter half of the models had significantly less violation than the former half, and the latter 10 spine models had significantly less time than the former 10 models	Using the model for training is helpful to improve the accuracy and speed of the trainee’s operation	The osseous feel may be different from the real pedicle	2018
Michael B. Gottschalk et al. Gottschalk et al. (2015)	To analyze the effect of surgical training using three-dimensional (3D) simulation on the placement of lateral mass screws in the cervical spine on either cadavers or sawbones	15 orthopedic residents, postgraduate year (PGY) 1–6, were divided into 3 groups. Group 1, control, did not receive any training, whereas Groups 2 and 3 received 3D navigational feedback as to the intended drill trajectory on sawbones and cadavers. Final test, all 3D images were deidentified and reviewed by a surgeon to determine trajectory accuracy	Groups sawbone and cadaver did better than the control group (*p* < 0.0001)	Training with 3D navigation significantly improved the ability of orthopedic residents to properly drill simulated lateral mass screws	Did not investigate whether training translated into improved operating room accuracy	2015
Dale J. Podolsky et al. Podolsky et al. (2010)	Assess the efficacy of a pedicle screw insertion simulator	28 residents from orthopedic surgery and neurosurgery were divided into patient and control groups. They both received standard training on pedicle screw insertion but the patient group received an additional 1-h session of training on the simulator. Qualitative feedback about the simulator was gathered from the trainees, and all pedicles screws physically inserted into the cadavers during the courses were evaluated through CT.	28% of the trainees who responded to the questionnaire and all fellows and staff surgeons felt the simulator to be a beneficial educational tool	The potential of the simulator to improve the teaching of difficult procedures, such as pedicle screw insertion	Comfort in using the simulator alone was limited	2010
William Clifton et al. Clifton et al. (2021)	A high-resolution segmentation and 3D-printing technique was investigated for the creation of a dynamic educational model	1.Investigation of segmentation and 3D printing technique validity through *ex-vivo* dynamic fluoroscopic assessment of a printed cervical spine model compared to parent patient DICOM imaging 2.Investigation of the educational value of a dynamic (malleable) cervical spine model compared to a static mode	The flexible 3D-printed do better in dynamic positioning and teaching the physiologic concepts of spinal canal change	Dynamic 3D-printed models is a cost-effective and novel educational tool	The absence of a simulated discoligamentous complex	2021

The related studies and bibliometrics results have shown interesting and meaningful results from many aspects. First of all, we noticed the substantial power of using 3D technologies in orthopedics medical education. We realize that they have an excellent promotion effect for medical beginners to learn orthopedics, especially for complex trauma, joint injury, spinal trauma and related biomechanical studies. As for 3D-printed models, students can quickly improve their skills in the learning process, while the 3D-printed models can achieve mass production (Park et al., 2018). And for complex acetabular fractures, 3D software seems to have a better ability to cultivate spatial thinking. On the other hand, 3D interactive software is better than traditional plane teaching in terms of objective learning improvement and subjective use satisfaction (Wang et al., 2019). And it can be foreseen that training with 3D navigation can quickly improve the skill of trainees. The feasibility and practicability of 3D navigation are verified by controlling variables (Gottschalk et al., 2015). And for young surgeons, especially beginners, if they could train and learn through 3D simulation training before the operation, it will bring them skill improvement with more confidence in the operation process. Although this gain seems to be weakening with the increase of employment time, it is of great significance for novices (Montgomery et al., 2020). On the other side, we have noticed that most of the subjective survey outcomes are very positive, showing that 3D technologies bring confidence and interest in a relative educational procedure to the students and are a promoter of teaching and learning. It is not only objectively outperforming other teaching methods but also plays a very positive role in changing the subjective attitudes of students towards learning. In other words, the application of 3D technologies can be regarded as the most ideal teaching aid among the currently available teaching aids. From another point of view, we have noticed that the majority of the subjective survey outcomes are overwhelmingly positive, representing that 3D technologies can bring more interesting and confident experience to students’ teaching process and are a promoter of teaching and learning. It is not only objectively outperforming other teaching methods but also plays a more than positive role in transforming the subjective attitudes of students towards learning. In other words, the application of 3D technologies can be regarded as the most ideal teaching aid among the currently available teaching auxiliary tools.

However, there are also limitations in the application of 3D technologies in orthopedic medical education as well as this study. First of all, 3D technologies are not perfect. Theoretically, no matter how realistic the materials and visual presentation are, there is no way to perfectly copy and display the bone, muscle, skin and other tissues of the human body, which is also the reason why human specimens cannot be replaced. Secondly, the initial production cost is also an important reason restricting its promotion. It requires scanning synthesis of a large amount of data. For some rare orthopedic diseases, few people are willing to pay a price for the 3D modeling and presentation. And for this study, it is inevitable that due to the different literature retrieval algorithms and the speed of literature update, it is difficult to collect and analyze all the literature in this field completely. The 3D technologies and orthopedic medical education-related publications are being continually published and cited, the bibliometrics results might change since we completed the analyses. Secondly, self-reference bias should also be taken into serious consideration and evaluation. We hope that in further research, with the update of literature management technology and retrieval methods in different databases, we will be able to obtain relevant data more accurately and get more convincing research results.

## Conclusion

This study makes a comprehensive bibliometric analysis of a large number of publications related to the application of 3D technologies in orthopedic medical education as well as orthopedic surgery training, presents and discusses the research results intuitively and visually. The research method is scientific and objective. In a word, the applications of 3D technology in orthopedic education are becoming more and more extensive and shows its effectiveness which have incomparable advantage over the traditional education mode.

## Data Availability

The original contributions presented in the study are included in the article/Supplementary Material, further inquiries can be directed to the corresponding authors.
